# “Temporal meaning-making of women's emotional experiences during the infertility treatment process: a qualitative study”

**DOI:** 10.1080/17482631.2026.2672214

**Published:** 2026-05-12

**Authors:** Nevra Karaca BIÇAKÇI, Ayşe Çalmaz, Birsen Altay

**Affiliations:** a Department of Nursing Public Health, Faculty of Health Sciences, Kafkas University, Kars, Turkey; b Hitit University İskilip Vocational School, Çorum, Türkiye; c Nursing Department, Public Health Department, Faculty of Health Sciences, Ondokuz Mayıs University, Samsun, Türkiye

**Keywords:** Infertility, biographical disruption, temporality, women's reproductive health, qualitative research, phenomenology.

## Abstract

**Purpose:**

This study examines women's emotional experiences during infertility treatment through the perspective of temporality, focusing on pre-diagnosis meanings, treatment experiences, and future expectations.

**Method:**

Using a descriptive phenomenological approach, semi-structured in-depth interviews were conducted with 19 women undergoing infertility treatment. Data were analyzed using Giorgi's method.

**Results:**

Infertility represents a 'biographical disruption' deeply affecting self-perception and social identity. Pre-diagnosis meanings attributed to motherhood deepen the trauma at diagnosis. The treatment process is an 'extended present' oscillating between hope and uncertainty, involving bodily objectification and intense social pressure

**Conclusion:**

Infertility is a multi-layered traumatic process. Health professionals should adopt a supportive care approach prioritizing women's temporal integrity and psychosocial well-being alongside medical outcomes.

## Introduction

Although infertility is not a health condition that directly threatens couples' lives, it is a process that challenges healthy living by negatively impacting the individual's psychological well-being and quality of life (Seymenler & Siyez, 2018). Entailing physical and psychosocial problems, infertility is defined as a life-altering experience that possesses cultural and religious dimensions and exposes the individual to unexpected stressors (De Berardis et al., 2014). During this process, women, in particular, may develop cognitive responses questioning their reproductive competence due to the association of the concept of womanhood with the roles of “wife” and “mother”; furthermore, societal gazes, implied remarks, and the meanings attributed to fertility influence women's self-perception (Pamuk & Karaca, 2023). From a societal perspective, although infertility is a challenging experience for both genders, the patriarchal social structure, the placement of the responsibility for childbearing primarily on women, and the equation of fertility with womanhood negatively affect the health of women experiencing the infertility process (Üner & Sunal, 2018).

According to the World Health Organisation (WHO), infertility is defined as the condition of failing to achieve pregnancy despite regular sexual intercourse without using any contraception method for one year (World Health Organisation, 2024). The increase in the prevalence of infertility is evaluated as a significant public health problem, although it shows regional differences worldwide (Çağlar & Yeşiltepe Oskay, 2020). According to WHO data, infertility is seen at a rate of approximately 17.5% globally, and it is reported that one in every six people is affected by infertility during their lifetime (World Health Organisation, 2023).

Women receiving a diagnosis of infertility and learning that they need treatment for pregnancy constitute the beginning of a process full of uncertainties. Physical, psychological, and social difficulties encountered during the treatment process create an experiential realm that is emotionally difficult to cope with for infertile women. Women experiencing the infertility process may feel lonely due to gender roles and social expectations, may not receive sufficient support from family and their environment, and may develop a negative self-perception (Deliktaş Demirci & Kukulu, 2021; Özdemir & Kaplan, 2021). It is reported that in this process, women experience stigmatisation due to childlessness, struggle psychosocially, and remain under the pressure of roles imposed on them (Deliktaş Demirci et al., 2024; Zorlu & Erbaş, 2023). Therefore, understanding the emotional states and thoughts of infertile women before, during, and after the treatment process is evaluated as an important necessity in terms of developing counselling services supporting women's health.

In this context, it is seen that for women, the infertility process is an experiential realm that does not consist solely of diagnosis and treatment stages, but changes over time and is re-interpreted. However, it is noteworthy in the existing literature that qualitative studies addressing infertile women's emotional experiences regarding the treatment process with a holistic approach in the context of past, present, and future are limited. For this purpose, this study, designed qualitatively, aims to contribute to the development of supportive and sensitive approaches towards women's health by examining, with a temporal perspective, the emotional experiences lived by infertile women at the diagnosis stage, throughout the treatment process, and in the context of expectations regarding the treatment outcome.

## Method

### Research design and purpose

Phenomenological research aims to reveal in detail the meanings attributed to these experiences by focusing on phenomena that individuals experience in daily life but are often not deeply aware of Titchen and Hobson (2005), Yıldırım and Şimşek (2006). The phenomenological approach contains different orientations, including interpretive, existential, and transcendental; in this study, the descriptive phenomenological research design, which is based on Edmund Husserl's understanding of transcendental phenomenology and systematised by A. Giorgi, was used (Giorgi, 2009). Giorgi's descriptive phenomenological approach aims to reveal the common meaning units in individuals' experiences and to describe the unchanging essences and structure of the experience (Denscombe, 2007). In this direction, in phenomenological research, experiences related to a specific phenomenon and the meanings attributed to these experiences are examined in depth (Patton, 2014). The aim of this study is to understand and describe, with a descriptive phenomenological approach, the emotional experiences lived by women during the infertility treatment process in the context of the diagnosis stage, throughout the treatment process, and expectations regarding the treatment outcome, within a temporal context extending from diagnosis to the treatment process and treatment outcome.

### Research questions

In this study, answers were sought to the following research questions:

- How do infertile women make sense of the phenomenon of becoming a mother within the framework of their own lives and the social context they are in?
- Which emotional experiences do women experience at the moment they receive the diagnosis of infertility?
- What are women's perceptions and emotional experiences regarding the treatment throughout the infertility treatment process?
- What are the emotional experiences accompanying women's expectations regarding the treatment outcome and the future?
- What are women's expectations from their spouses, families, social environments, and healthcare providers during the infertility process?

### Participants and sampling

The participants of this study consist of women diagnosed with infertility who are in the active treatment process. Purposive sampling was employed in the research, and participants were recruited through the snowball sampling method. Inclusion criteria were: being diagnosed with infertility at least two years ago, having a history of treatment at different centres or with various physicians while currently undergoing treatment at a medical faculty hospital, and volunteering to participate in the study. Due to the private and sensitive nature of the infertility experience, snowball sampling was preferred for reaching participants. The recruitment process was initiated with the first female participant who met the inclusion criteria and was reached through the referral of a specialist physician at the relevant medical faculty hospital. The sample was expanded by reaching other women within the social networks of the initial and subsequent participants who shared a similar treatment history and agreed to be interviewed. A total of 19 women were included in the study. Data collection was concluded with 19 participants upon reaching data saturation and “information power” (Malterud et al., 2016), which justifies sample size in qualitative research, as responses in participant narratives began to repeat.

### Data collection

In this study, data were collected through semi-structured interviews in accordance with the descriptive phenomenological approach. The interview questions were structured to reveal how the infertility experience is temporally made sense of by women. Accordingly, the questions were created to cover four basic temporal dimensions: meanings attributed to becoming a mother prior to the infertility diagnosis, emotional reactions experienced at the moment of learning the infertility diagnosis, lived experiences throughout the treatment process, and future-oriented expectations regarding the treatment outcome and social support. Interviews were conducted in environments where participants felt most secure and comfortable, ensuring full confidentiality to facilitate the discussion of a sensitive topic like infertility. Accordingly, the choice of interview venues was left to the participants; some interviews took place in quiet, small classrooms used by assistant physicians within the hospital setting, some in suitable and isolated areas at participants' workplaces, and others directly in the participants' own homes. During the audio-recorded interviews conducted with participant consent, participants were allowed to recount their experiences freely in their own words; probing questions were utilised when necessary to deepen the exploration of these experiences. Although the duration of each interview varied depending on the participant, the average length was approximately one hour.

Prior to the data collection process, the procedure of epoché (bracketing) was implemented to ensure phenomenological integrity. The researchers made their academic knowledge in the field of women's health, clinical experiences regarding infertility, and personal preconceptions about the concept of “motherhood” visible by recording them in a reflexive journal and consciously suspended them. Thus, the interviews with participants were approached not through a medical expert lens, but with an open and curious mind, as if hearing the phenomenon for the first time.

### Data analysis

In the analysis of the data obtained in this study, the descriptive phenomenological analysis method developed by A. Giorgi was used (Giorgi, 2009). The aim in phenomenological analysis is to reveal the meanings common in individuals' lived experiences and to describe the unchanging essences and structure of the experience. Accordingly, the audio recordings obtained from the interviews were transcribed verbatim, and the texts were prepared for the analysis process. A holistic familiarity with the data was ensured by the researcher by reading the texts repeatedly. The analysis process was carried out in accordance with the four basic stages proposed by Giorgi. In the initial stage, the reflexive attitude initiated during the data collection process was maintained, and the procedure of bridling Dahlberg (2006), was meticulously applied throughout the analysis. While transcribing the participants' expressions and constructing meaning units, a conscious effort was made to avoid hastily labelling the emotional experiences recounted by the women with concepts from existing clinical or psychological literature. Through this bridling attitude, which deliberately slows down the process of understanding, the data was prevented from being confined within predetermined theoretical frameworks; instead, meaning units were allowed to emerge in their natural flow, originating entirely from the women's own words and their lived worlds. Subsequently, during the stage of phenomenological reduction, participant narratives were examined to determine meaning units related to the experience, and the texts were partitioned into distinct meaning units in accordance with the nuances of the expressions. In the process of imaginative variation, the relationships between these meaning units were analysed to reveal the structural themes of the experience. In the final stage, meaning units derived from participant profiles were synthesised to describe the invariant essences and structure of the infertility experience. Throughout the analysis process, participant experiences were addressed within a temporal context: meanings attributed to motherhood prior to the infertility diagnosis (past), experiences during the diagnosis and treatment process (present), and expectations regarding treatment outcomes and social support (future). Participant quotes and phenomenological structural components were translated from Turkish into English using the back-translation method to preserve the essence and psychological depth of the data. In the first stage (forward translation), the original Turkish texts were translated into English by a bilingual expert with native-level English proficiency. In the second stage (back-translation), these English texts were translated back into Turkish by the researcher, who possesses a comprehensive command of the cultural and psychological context of the data. In the final stage, the original Turkish texts and the back-translated versions were meticulously compared by the researcher. Rather than a literal translation, a phenomenological and meaning-oriented approach was adopted throughout this process. The translator and the researcher collaboratively discussed and reached a consensus on emotionally charged expressions, cultural metaphors, and infertility-specific psychological language (e.g., sense of incompleteness, traumatic grief) that carried a risk of being lost in translation. Consequently, English expressions were revised to fully reflect the original emotional tone and psychological reality of the women's lived experiences.

**Trustworthiness** in accordance with the nature of qualitative research and the descriptive phenomenological approach, Lincoln and Guba's (1985) strategies of credibility, dependability, confirmability, and transferability were adopted to ensure the rigour of the study. **Credibility** was established through the procedures of *epoché* and *bridling* applied during data collection and analysis, allowing the researchers to focus strictly on the participants' lived experiences. **Dependability** was ensured by strictly following Giorgi's (2009) systematic four-stage analysis method. To maintain ontological consistency with phenomenology, the study avoided “interrater reliability” metrics; instead, an interpretive consensus focusing on the essence of the phenomenon was achieved through continuous researcher reflexivity. **Confirmability** was provided by supporting the structural constituents with rich, direct participant quotations to demonstrate that findings emerged from the data rather than researcher bias. Finally, **transferability** was achieved through “thick description,” providing detailed information about the participants, the research context, and the sampling process to allow for contextual comparisons.

### Ethical considerations

Ethics approval was obtained from a university's social and humanities research ethics committee for the study. The research process was conducted after the ethics committee approval was obtained. Detailed information about the purpose, scope, and interview process of the study was given to all participants participating in the research; **written** informed voluntary consent was obtained from the participants. Participation was based entirely on voluntariness, and it was stated to the participants that they could withdraw from the study at any stage they desired. Participant confidentiality and privacy were observed at all stages of the research, and data obtained during the interviews were used solely for scientific purposes. In order to protect participant identities, names were not used in the presentation of the findings, and each participant was defined through codes. The obtained data were stored in a secure environment and were not shared with third parties.

## Results

The mean age of the 19 women participating in the study was 31.47 ± 3.48 (min: 27, max: 38) years, and their mean duration of marriage was 8.15 ± 5.13 (min: 3, max: 14) years. The participants' mean age at first marriage was 23.26 ± 4.16 years. Regarding educational status, 8 women had a higher education degree or above, 6 were high school graduates, and 5 were middle school graduates. Furthermore, it was determined that a vast majority of the participants were not employed in an income-generating job. According to their income status, it was found that the income of the majority (*n* = 10) was equal to their expenses. A large proportion of the women (*n* = 15) did not have any chronic diseases, and similarly, a vast majority (*n* = 15) reported no additional infertility-related problems in their sexual lives.

In the data analysis process, the steps determined by Giorgi's (2009) were followed. The raw data expressed by the participants with the “natural attitude” were examined by the researcher with a phenomenological perspective and re-expressed in psychological language (transformation). Subsequently, these transformations were gathered under the structural constituents reflecting the invariant essence of the infertility experience. In accordance with Giorgi's approach, these structural constituents are not independent themes; rather, they are elements within an inseparable “part-whole” relationship that constitute the general structure of the experience. A sample cross-section regarding the analysis process is presented in Table I.

**Table I.** An example of descriptive phenomenological analysis of the infertility experience in a temporal context.

Sıra	Participant statement (raw data/meaning unit)	Psychological transformation (researcher's interpretation)	Constituents of the structure	Temporal context
**1.**	**(P-3)** “To become a mother means to multiply, to be complete, to be whole. Just thinking of oneself as a mother brings a smile to one's face. Not being able to become a mother is like a deficiency, like being left half.”	The participant defines motherhood as a fundamental element ensuring her own existential integrity, beyond a biological function. The state of childlessness is experienced as an ontological sense of “incompleteness” and “deficiency” felt within the self, rather than a physical absence.	**Existential Completion and the Sense of “Being Left Half**	**Past** (Pre-diagnosis Meaning-Making)
**2.**	**(P-2)** “When I first learned about it, I felt sad as if I had been given the news of a relative's death. I felt very helpless. Why did God give me this punishment? Is being infertile my test?”	The moment of diagnosis is perceived by the participant as a shock equivalent to a mourning process (news of death) and a life crisis. The medical condition is detached from rational causes, attributed to a metaphysical causality (punishment/test), and transforms into an intense sense of guilt.	**Biographical Disruption and Traumatic Grief**	**Present** (Moment of Diagnosis and Emotional Reaction)
**3.**	**(P-19):** “I have memorised how the treatments are by now, we keep doing the same things. I know my tests, I know the medications... They are like machines, they don't even look at our faces.”	The treatment process has turned into a mechanised and routinized cycle for the participant. The perception of the body as a medical object and the absence of an emotional bond established with healthcare personnel deepen the sense of alienation in the process.	**The Objectified Body and Mechanisation**	**Present** (Treatment Process)
**4.**	**(P-12):** “I am not very hopeful about the result of the treatment, I experienced disappointment every time... But I still have to try, waiting is very hard.”	The expectation regarding the future is a paradoxical situation stuck between hope and hopelessness. Although past failures weaken the belief regarding the future, the social “imperative” of motherhood forces the participant to remain in action (in treatment) despite the hopelessness.	**Uncertainty Between Hope and Hopelessness**	**Future** (Expectations)

In this study, the infertility experience was examined within a temporal structure based on participant narratives. As a result of the analysis, it was observed that for women, infertility is not limited to a single moment; rather, it is a multi-layered realm of experience shaped by meaning-making extending to pre-diagnosis, the emotional ruptures at the moment of the formal medical announcement of the diagnosis by a physician in a healthcare facility, experiences lived throughout the treatment process post-diagnosis, and expectations regarding the treatment outcome. It is noteworthy in the narratives of the participants that the infertility experience is made sense of in a manner intertwined with womanhood, motherhood, and social roles; and that while this meaning-making changes over time, certain emotional and cognitive structures show continuity. In this context, the findings reveal how women experience the infertility process through meanings shaped in the light of past experiences and social acceptances, intense emotional experiences pertaining to the present, and hope, uncertainty, and expectations regarding the future. In the presentation of the findings, meaning units derived from participant narratives and the structural characteristics of the experience indicated by these meaning units were taken as the basis. Considering the temporal flow of the infertility experience, the findings were described within the framework that the meanings attributed to becoming a mother before diagnosis, emotional reactions to learning the infertility diagnosis, perceptions and expectations regarding the treatment process and outcome, and social support expectations constitute a realm of experience showing continuity starting from pre-diagnosis through the moment of diagnosis, the treatment process, and expectations regarding the treatment outcome. In each section, how women expressed their experiences in their own words and how they attributed meaning to these experiences were made visible through direct quotations.

**Figure 1.**
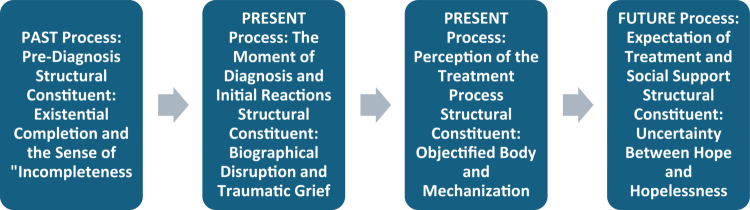
Temporal general structure and structural constituents of the infertility experience.

Although the questions followed a specific temporal sequence due to the semi-structured nature of the interviews, the general phenomenological temporal structure and the psychological constituents comprising this structure (e.g., traumatic grief, objectified body) emerged naturally from the data through the analysis of direct participant expressions rather than from the researcher's pre-construction. This temporal general structure and its sub-constituents derived from the data are visualised in Figure 1.

### Meanings attributed to becoming a mother before diagnosis

In participant narratives, becoming a mother emerges as a realm of experience that extends to before the infertility diagnosis and holds a central place in women's lives. The thought of becoming a mother is expressed in the participants' statements not merely as a biological state, but as an experience made sense of together with how the woman positions herself, how she is associated with womanhood, and how she is perceived within society. In this period, becoming a mother is associated at the individual level with feelings of completeness, worthiness, and gaining existence, and is also expressed within a framework of meaning intertwined with social acceptance, respectability, and roles of womanhood. The participants' narratives demonstrate that becoming a mother carries strong meanings regarding both the women themselves and their positions within society in the pre-diagnosis period.

Some participants define becoming a mother as an experience that completes the meaning of life and happiness. This meaning-making indicates that becoming a mother is viewed as a state that adds value to the woman's existence.

*"I think it’s the most beautiful feeling in the world. Just imagine, a child of your own, it’s a magnificent thing. Happiness, something like a miracle. To be a mother is existence."* (P1, 28)

In participant narratives, the experience of becoming a mother is also associated with feelings of multiplying, being whole, and belonging to someone. For some women, becoming a mother carries the meaning of a part of themselves continuing in life.

*"To be a mother means: to multiply, closeness, to be complete, to be whole, to be accepted, it means someone is a part of you. Even just thinking about being a mother makes one smile."* (P3, 36)

In participant narratives, becoming a mother becomes visible not only as an individual desire but also as a role socially attributed to the woman. In these narratives where becoming a mother is identified with womanhood, it is expressed that the inability to become a mother creates a sense of deficiency.

*"I don't know what it feels like to be a mother. I didn't have a mother either. I never knew my mother. Motherhood means a blood bond. Not being able to be a mother is a deficiency. The sense of belonging doesn't develop when you aren't a mother."* (P6, 35)

Some participants define becoming a mother as one of the fundamental requirements of marriage and life; and associate the existence of a child with the purpose of life.

*"Having a child is the fundamental reason for marriages. Why would people even get married if there won't be a child? A child brings happiness; a person becomes just like a tree bearing fruit. If you don't have a child, it means your hands are empty, you have no occupation, you have no purpose."* (P7, 34)

In participant narratives, the experience of becoming a mother is expressed together with emotions such as happiness, love, future, compassion, and devotion, and is placed in a central position in the woman's life.

*"To be a mother means happiness, future, love, peace, compassion, devotion, existence, beauty."* (P10, 29)

Some participants, on the other hand, associated becoming a mother with a warm home, becoming a family, and womanhood.

*"To be a mother means a warm home, becoming a family, being a woman, being female."* (P17, 32)

Participants expressed their experiences regarding how becoming a mother is construed in society in detail. In participant narratives, becoming a mother is expressed as a fundamental criterion determining the woman's worth, respectability, and position by society. It is indicated that women who are not mothers are marginalised, devalued, and stigmatised in society.

One participant expressed society’s attitude towards women who cannot become mothers as follows.

*"Society marginalises women who cannot become mothers; they pity the woman with words like ‘poor thing.’ I get very angry when this happens. There are two things in society that make a woman valuable: the first is having a child, the second is earning money. When these are missing, it means you have no worth as a woman in anyone’s eyes. If she can give birth to a child, the woman is considered a perfect woman."* (P6, 35)

Another participant expressed that becoming a mother is directly associated with receiving respect and value in society, whereas women who are not mothers are devalued, with these words.

*"In our society, if you become a mother, you receive respect, you are valued, your words are listened to. If a woman is not a mother, you are considered useless, like a dried-up tree. You are evaluated as a half-woman who is good for nothing."* (P7, 34)

Some participants noted that women who are not mothers are constantly criticised in society, that different responsibilities are imposed upon them, and that they are devalued.

*"From society’s perspective, to be a mother means: to be accepted, to be like everyone else, to be respected, to be completed. Someone who isn't a mother is stigmatised; they look at her as lacking, they treat her as if she isn't a woman. To society, being a 'half-woman' means... if you don't have a child, those around you start calling you 'empty-handed,' they say she has no occupation. For that reason, they want women who aren't mothers to do every kind of work; I mean, you are at everyone's service, they treat you like a slave. Everyone expects you to do work because you aren't a mother and you have no occupation. They act as if the one who isn't a mother has no responsibilities either. Society treats the woman who isn't a mother badly; first of all, they label her as barren."* (P15, 38)

It is clearly observed in participant narratives that becoming a mother is associated with power, health, and respectability in society, while women who are not mothers are humiliated and excluded.

*"From society's perspective, to be a mother means power, being capable, being healthy. In this society, a woman who doesn't have a child is looked at like a **rotten egg**. If you don't have a child, they say 'this one is barren,' they say 'what a pity for her husband.' Because childlessness means unhappiness, worthlessness. When you become a mother, a woman’s word is listened to, the woman is loved, no one can say a word against her, no one can humiliate her. To be a mother is like **a reign**. If you don't have a child, you become a woman like a servant, a slave behind everyone else."* (P17, 32)

These findings reveal that becoming a mother constitutes a strong and multi-layered framework of meaning for women prior to the infertility diagnosis; and that this framework is shaped in line with women's individual experiences and the meanings attributed to becoming a mother by the society they live in. In participant narratives, becoming a mother becomes visible not only as a personal desire or life goal, but also as a realm of experience associated with social acceptance, respectability, and roles of womanhood. In this regard, in women's narratives during the pre-diagnosis period, becoming a mother emerges as a fundamental realm of experience that forms the emotional foundation of the experiences regarding diagnosis, treatment, and the future to be lived in the subsequent processes.

### Initial emotional reactions to learning the infertility diagnosis

In participant narratives, the moment the infertility diagnosis is learned emerges as an experience that interrupts the ordinary flow of life and creates a profound emotional turmoil for women. The moment of diagnosis was experienced not merely as the sharing of medical information, but as a breaking point where women's thoughts regarding themselves, their futures, and their expectations of becoming a mother were simultaneously turned upside down. In this process, astonishment, fear, sadness, helplessness, hopelessness, and intense bodily reactions were experienced together.

In some narratives, learning the diagnosis is expressed along with an unexpected shock and mental confusion. Women have expressed in detail the astonishment and the state of momentary freezing that arose from encountering such a diagnosis at a time when they felt healthy.

*"When I first learned that I needed treatment to become pregnant, I went into shock, I think. I was not expecting such a result at all. I thought the doctor would say you are healthy, you will have a baby. I was astonished, I was scared, I was very sad, and I became unable to understand what the doctor was saying. I don't exactly know what I should think at that moment. I guess I first thought it was something simple, that I could become pregnant with a small treatment, that the problem would be solved, but on the other hand, I started thinking, 'what if it doesn't happen?' As you can imagine, one good and one bad thought was passing through my head. I looked at my husband's face at that moment; he was also astonished. I remember saying, 'Give me a glass of water.'"* (P4, 29)

In other narratives, the moment of diagnosis is depicted along with an intense feeling of loss and bodily collapse. In this experience, infertility was perceived as a life possibility taken away from the woman and was associated with a deep sense of devastation.

*"When I heard that I wouldn't be able to have children without treatment, I felt like I had collapsed, I felt pain, I was very, very sad, as if something had been taken away from me. My heart felt tight. I felt like the world had crashed down on me. My periods were irregular even before I got married; all those years, I kept saying, 'What if I don't have a child?' I thought what I feared had come true. When the doctor said, 'Don't worry, treatment has advanced,' I started crying. I started thinking that difficult times were ahead of me. I immediately wanted to go outside and take a breath."* (P5, 27)

In some narratives, learning the diagnosis was construed as a devastation equivalent to news of death. In this process, not only sadness but also intense helplessness, guilt, religious questioning, and concerns regarding the marital relationship were experienced together.

*"When I first learned it, I was so sad, it was as if they had given me the news of the death of a close relative. I felt very helpless. I started thinking, 'What am I going to do now?' Will I not have a child? Will there be no one in this life who calls me Mother? Why did God give me this punishment? Is being barren my test? I felt like my tongue and palate were dry. How am I going to tell this bad news to my husband? When I left the hospital, I walked for hours; I didn't even take the bus. When I got home, I cried a lot, and I'm still crying. I got incredibly angry, tense, I trembled, I thought I was suffocating. I got married without my family's permission. My husband's family still doesn't want me. I thought they would want us to get divorced. I immediately went outside, then I sat under a tree and started crying because I was barren. For a few months, I lied to everyone; I told lies that the doctor said, 'You are very good, you have no problem, you will have a baby.'"* (P2, 27)

Among the reactions to the diagnosis are denial, redirecting responsibility to someone else, and questioning the validity of the diagnosis. In these narratives, women are seen to enter an intense search process before accepting the diagnosis.

*"When I first learned it, I was very surprised. I had no health issues, my periods were regular, and I even thought it was too early for a child. I used medication for a long time to prevent pregnancy. When I heard that I needed treatment to become pregnant, I cried a lot; I couldn't accept it. Initially, I thought the fault wasn't mine, it was my husband's. I was healthy. I went to various other doctors thinking there might be a mistake. I researched a lot. Once I was convinced that the problem was mine, I started going back to the first hospital for treatment. I have a close friend. She has three children. She can get pregnant whenever she wants. I was very jealous of her. I didn't see her for a long time due to jealousy. It seems when a person can't have something, they become jealous of their closest friends who do have it."* (P11, 29)

For some women, learning the diagnosis has combined with past life experiences, bringing with it a profound feeling of loneliness and anxiety about the future. In these narratives, infertility was expressed as a new burden added to existing fragilities in life.

*"When I learned that I wouldn't have a child without treatment, the first thing that came to my mind was that I would be left alone in this world. I felt helplessness, I felt pain and loneliness. I grew up without a family; I thought there would never be anyone of my blood, of my flesh. I withdrew into myself; I didn't talk to anyone for a long time. Learning that I was barren was a huge disappointment for me. I thought, 'I couldn't get an education, I didn't have a job, and now I won't have a child either.' I didn't know what I should do for a long time."* (P16, 35)

These findings reveal that the moment of learning the infertility diagnosis is not merely a medical information process for women; it is experienced as a profound **realm of experience** that **simultaneously shakes** the meaning of life, future expectations, and perceptions of womanhood. In participant narratives, the moment of diagnosis is depicted as a **breaking point** that begins with astonishment and denial, and deepens with intense sadness, helplessness, bodily reactions, and feelings of loneliness. This experience causes women to re-evaluate their thoughts about themselves, their relationships, and the future; it also constitutes the **emotional foundation** of the experiences to be lived throughout the treatment process. In this regard, the moment of diagnosis emerges as a **decisive threshold** in women’s lives during the infertility process.

### Perceptions regarding infertility treatment and expectations for treatment outcome

Perceptions regarding infertility treatment and expectations for the treatment outcome emerge in participant narratives as a **realm of experience** that starts after the diagnosis and is **intertwined with future thoughts**, experienced temporally within an **“extended present.”** While describing the treatment process, participants simultaneously express their experiences concerning the ongoing procedures, medications, and healthcare services, and their hopes, hopelessness, and expectations regarding the outcome of this process.

Perceptions regarding treatment are shaped in some participant narratives alongside long-term attempts and recurring failures. This situation brings with it a strong sense of hopelessness regarding whether the treatment will work.

*"I don't think the treatments will work anymore; I've used a lot of medication for years. We need to have IVF. My husband doesn't want it. I have no hope at all regarding the outcome of this treatment either. There's nothing we haven't tried."* (P9, 36)

Another dimension highlighted in the narratives regarding the treatment process is the experience of the process as a **complex, difficult to understand, and uncertainty-filled structure** by the participants. Information received from different physicians, numerous treatment options, and the experiences of other women in their environment **further complicates** this confusion.

*"The doctor said there were too many treatment options, he explained the details of the treatment, I went to other doctors as well, and they said similar things. All of this is very complex, I find it difficult to understand. I researched a lot, I read a lot, I resorted to many different methods, yet it still feels complicated. I know other women undergoing treatment, I talked to them extensively, we have similar problems, but the medications given are different, I don't know, sometimes I just get confused."* (P12, 32)

This confusion also **directly affects** the participants' expectations regarding the treatment outcome. In some narratives, every new attempt brings with it disappointment and the **emotional burden** created by waiting.

*"Frankly, I am not very hopeful about the outcome of this treatment; I was disappointed every time, I was devastated every time they said 'unfortunately.' The worst part of the treatments is waiting for the result; time just doesn't pass. I have no hope regarding the outcome of this treatment."* (P12, 32)

Some participants, however, define the treatment process as a **series of procedures** that must be fulfilled, rather than how they **construed** it emotionally. In these narratives, treatment becomes a **routine obligation** rather than an active process of hope.

*"What should I think about the treatment, I really don't know. They give me medication, I take it precisely on time, I do everything the doctors tell me to."* (P13, 33)

The same participant's continuing statement demonstrates how the expectation regarding the treatment outcome is intertwined with hopelessness.

*"Honestly, with all this fate involved, I have no hope that I will get pregnant."* (P13, 33)

The **necessity of discreet execution** (conducting the treatment secretly) of the treatment process was expressed by some participants as a factor that both **limits treatment options** and transforms the process into a more **burdensome and repetitive experience**. This situation causes the treatment to be experienced over time as a **memorised and cyclical process** sustained within the same loop.

*"My husband's family still doesn't know that I'm receiving treatment. For that reason, I cannot get advanced treatments; they only prescribe medication, and I take the medicine secretly. I have memorised how the treatments work now; we just keep doing the same things. I know my test results, I know the medications, sometimes I use/take more than what the doctor prescribed."* (P19, 30)

These narratives demonstrate that infertility treatment is construed by participants not merely as an ongoing medical process, but also as a **realm of experience** lived in **continuous contact** with expectations regarding its outcome. While perceptions of treatment are shaped by uncertainty, confusion, and repetitive attempts, expectations for the treatment outcome are mostly expressed within an emotional state **alternating between hope and hopelessness**. In this respect, infertility treatment emerges in participant narratives as a **multi-layered experience** lived within the **“extended present,”** starting after the diagnosis and extending into the future.

### Social support expectations

Social support expectations emerge in participant narratives as a **realm of experience** that begins **starting from the infertility diagnosis** and continues throughout the treatment process, constantly **re-shaping** alongside future expectations. In this context, social support is expressed not as a need specific to a particular moment, but as a need that **spreads across the entire infertility process** and is continually **re-experienced** within relationships established with the spouse, family, social environment, and healthcare providers.

Participants expressed that they primarily expect **understanding and patience** from their spouses, and mostly request **financial support and a non-interfering approach** from their families. At the same time, questions and guidance received from the social environment are often seen to be **experienced as pressure** rather than support.

*“I would only ask my husband to be patient; if the problem were with him, I would be understanding towards him. Sometimes he says we are struggling for nothing, and that upsets me greatly. My expectation from my family is financial; I want to get better treatment in better places. Neither my family nor my husband's family helps at all. People around me constantly ask, 'What did the doctor say, how is the treatment going, what percent chance do you have?' and so on, constantly asking. They give advice, every person offers an opinion, saying 'she did this, he did that, you should do it too,' and it gets on my nerves; it's better if they don't interfere. The health personnel, in general, are good, they help, but I wish hospitals would do everything for free.”* (P1, 28)

Some participants, however, stated that they have **life stories** where family support was either non-existent or quite **limited**, and for this reason, they **directed their expectations** more toward healthcare personnel.

*“I have no expectations from my husband; I want us to have a child, I would leave if we don't have a child, no problem. I never had a family, so naturally, there was no support from family. I grew up in a state dormitory, I don't have much of a social circle. Only my husband's family is there, and I don't expect anything from them either. I expect the healthcare personnel to be kind, and I would also like them to answer every question openly. They answer without looking at you, that is very upsetting.”* (P8, 31)

While the existence of spousal support is **expressed as carrying the process together** in some narratives, interventions from family and the social environment are seen to constitute an **emotional burden** on the participants. Expectations regarding healthcare personnel are mostly voiced through communication, clarity, and a **humane approach**.

*“My husband actually supports me a lot, but he is also very upset himself. He really wants to have a child, even a son. We never used any contraception; we have always wanted a child from the start. My husband has no siblings, so he really wants to have a child. I don't expect support from my family; it's enough if they don't get on my nerves. They hear things from this person and that person and come and tell me; so-and-so got divorced, this one adopted, etc., etc. I know they are afraid too, but this is not how you offer support. I tell them to be quiet, I tell them not to say anything to me, not to ask anything. I don't have any expectations from my social circle either; it's enough if they don't ask questions about children. I feel like shouting at them, I feel like telling them to mind their own business. The healthcare personnel are like robots, they are all emotionless, annoying. For example, when they take blood, they don't even look at my face; they chat with their friends. 'Sit down madam, stand up madam,' that's all. The doctors also won't say anything unless we ask questions; they are better in private practice, at least they explain things. For this reason, I don't have much trust left in the healthcare personnel either.”* (P10, 29)

In some participant narratives, the spouse's hopelessness stands out as a factor that reduces motivation for the treatment process. The **lack of support** expected from family and the social environment is expressed along with **emotional offence** and **anger**.

*“My husband always says that if we can't have a child, we will adopt. That makes me very angry. He doesn't say, 'Let's try every way,' he seems more hopeless than I am, yes, more hopeless than I am, I wish he were hopeful and said, 'Let's go all the way.' My mother's family is financially comfortable, treatments are expensive, if they gave us financial support, we would try IVF two or three times a year. I don't expect any support from the people around me, but they ask too many questions. Like, 'Why don't you have a child?' They shouldn't ask questions, I don't even want support. Regarding the healthcare personnel, we've had many procedures done; they do the procedure and don't give any explanation. When you ask something, they say, 'Ask your doctor,' classic healthcare workers, you know.”* (P14, 29)

Some participants stated that they experienced the complete **absence of spousal support** along with **isolation** and **financial constraints**. This situation indicates that the failure to meet social support expectations **exacerbates the process**.

*“My husband doesn't support me at all. I tell him we have enough money, let's get more advanced treatment, but he doesn't want to. Because my husband already has two children, he has become a father, he doesn't care about me. I used to work, he wanted me to quit my job, and now he doesn't even give money for treatment. My husband is 53 years old, he says, 'What will we do with a child at this age?' I really wish my husband would support me, but unfortunately, I usually go to treatment alone. I am very angry at my family, very offended, they don't support me at all. I asked for financial support from my family, but they said my husband had money and didn't provide any support. They say it's not right for me to have a child at this age. They say my siblings have children, and I should take care of them, that loving them is enough. The people in my social circle are generally very ignorant, they gossip a lot, so I don't talk about these issues with them, and I even prefer they don't ask me questions. I am very angry at them, sometimes I feel like cursing them. The healthcare personnel are very indifferent, I wish they would explain everything about the treatment openly. I can't even fully understand what they are saying. Even the doctor usually just examines me and prescribes medicine, he doesn't give much explanation.”* (P15, 38)

In some narratives, the existence of spousal support, even if **limited**, **makes the process more bearable**, while expectations from healthcare personnel are seen to be heavily **concentrated on the communication dimension**.

*"I don't get much support from my husband; he doesn't really believe what the doctors say, and I would expect him to believe the doctors. My husband says that God gives the child, what do the doctors know, and he doesn't even want to come to the hospital. My family and social circle have generally been supportive of me. The most important thing I want from the healthcare personnel is for them to explain everything about the treatment openly. I wish they would be a little more helpful and give clear answers to questions; I can't understand what they are saying, and when I ask a second question, they look at me as if they are angry. They are like machines."* (P18, 27)

Some participants stated that they received **emotional support** from their spouses, while expressing that **curious questions** from the social environment were experienced as a **bothersome experience**.

*"I always wanted my husband to be understanding towards me during the treatment process, and he truly was. He always gives support. I don't share the treatment process much with my family because they get too upset. In my social circle, however, I encounter things I really hate; they constantly ask curious questions, **as if it were their duty**. This problem is our problem, it doesn't concern them. Their only desire is to satisfy their curiosity, and it makes me nervous. Regarding the healthcare personnel, I don't have many expectations from them; I just wish they would be a little more cheerful, nothing else, they talk to us as if they are scolding us."* (P19, 30)

These findings reveal that social support expectations are not specific to a certain time in the infertility process but constitute a **realm of experience** demonstrating **continuity**, extending from the diagnosis to the treatment process and future expectations. In participant narratives, social support is experienced sometimes as a **supportive factor** that carries the process with its presence, and sometimes as an element that **increases the emotional burden** with its absence.

## Discussion

This phenomenological study elucidates the emotional experiences of women undergoing infertility treatment within a coherent temporal framework structured around past meaning-making processes, present treatment-related challenges, and future-oriented expectations. The findings demonstrate that infertility, for women, is not merely a condition of medical insufficiency; rather, it constitutes a “biographical disruption” that profoundly destabilises their sense of self, social identity, and anticipated life trajectory. Notably, the infertility experience does not begin at the moment of diagnosis; instead, it is shaped by deeply rooted meaning-making processes that extend into the pre-diagnostic period. Throughout the study, the concepts of biographical disruption and master status were employed not as fixed or pathologizing labels attributed to women; rather, they were treated as contextual lenses that render visible the depth of their lived experiences and meaning-worlds in the face of social pressures.

The study's findings regarding the meanings women attributed to motherhood before the diagnosis reveal that motherhood, beyond a biological function, is a **central vehicle** for **“sense of completion”** and **“gaining existence”** in the **construction of female identity**. The participants' description of motherhood using strong metaphors such as **“dignity”** and **“dominance”** aligns with the literature in cultures like Türkiye, which exalt fertility and have a traditional social structure. Remennick (2000), states that in pronatalist societies, motherhood is viewed almost synonymously with womanhood; therefore, childlessness means women are deprived of the most fundamental element of their gender identity and personal integrity. In this context, the participants' characterisation of themselves as **“incomplete”** or **“half”** indicates that infertility ceases to be a disease and becomes a **“dominant status”** that defines the person's entire self. The literature emphasises that this degree of intertwinement between motherhood and female identity is a **social and cultural construction** rather than a biological process (Beauvoir, 1970; Bhatti & Jeffery, 2012). Sarı (2014), states that the patriarchal system presents motherhood to women as a natural, sacred, and undeniable **“destiny”**; thus, the inability to have children leads to a deep feeling of inadequacy in women. In the current study, participants' association of motherhood with **“productivity”** and **“gaining value”** also supports this view (Parlak & Tekin, 2020). Therefore, becoming a mother is experienced as an **obligatory path** to gaining **social acceptance and status**, rather than an **individual desire**. These strong construals shaped in the pre-diagnostic period also determine the **severity of the trauma** at the moment the infertility diagnosis is learned. In this context, where social acceptance is achieved through motherhood, an infertility diagnosis signifies for the woman not only the absence of a baby but also the **loss of the expected social status** and **“stigmatisation”** (Dierickx et al., 2018; Whiteford & Gonzalez, 1995). This situation demonstrates that infertility counselling should aim not only to manage the medical process but also to repair the woman's **shattered social identity and self-esteem**.

The study findings reveal **the formal medical announcement of the infertility diagnosis** as a traumatic 'life crisis' that interrupts individuals' biographical flow, threatens their self-esteem, and future plans. The astonishment, fear, helplessness, and hopelessness experienced by participants upon first hearing the diagnosis align with the literature that defines infertility as a **complex crisis** (Beji, 2001; Boivin et al., 2001). Infertility is a **multi-dimensional process** that profoundly affects not only individuals' reproductive ability but also their social lives, marital relationships, and body image. However, the way this crisis is experienced can vary according to cultural factors, family structure, and individuals' perspectives on pregnancy, as also stated by Bayraktar (2018). The initial reactions given at the moment of diagnosis are generally described in participant narratives as a state of **“shock”** and **“loss of self.”** This situation parallels the denial, **psychological breakdown**, and disbelief of the sorrowful truth experienced by couples when they learn they cannot have children (Keskin & Gümüş, 2014; Koçak & Büyükkayacı Duman, 2016). Oltuluoğlu and Günay, 2014, similarly state that anger, guilt, and an **intense psychological devastation** are seen in individuals immediately following the diagnosis.

The **sudden and intense emotional upheaval** observed in our study is also related to a deep **overwhelming feeling of guilt** stemming from women's perception of infertility not just as a medical problem but, in Remennick (2000), words, as a **“stroke of fate”** or a **“punishment.”** As the process continues, this state of shock gives way to anxiety, depressive symptoms, and an inability to face reality. The **mental confusion** experienced by women when describing their unsuccessful treatment stories in the study aligns with (Kissiwaa & Fouché, 2024), finding of **“anxiety blurring the mind.”** Women exhibit depressive symptoms and high anxiety while struggling with feelings of failure and inadequacy during the infertility process (Deliktaş & Kızılkaya Beji, 2012; Güz et al., 2003). However, when approached from a phenomenological perspective, the profound sadness, anxiety, mental preoccupation, and grief experienced by these women should be understood not as clinical psychiatric disorders (pathologies), but rather as deeply human, natural, and situational responses to an existential threat, uncertainty, and successive losses (Aktürk, 2006), It emphasises that this process initiates an unusual form of grief (mourning). Indeed, the “grief” experienced by the participants, despite the absence of a visible loss, manifests as a profound sadness and exhaustion arising from the loss of the hoped-for baby. (Draye, 2004). Remennick (2000), explains this situation as the effort to achieve pregnancy becoming an **“obsession”** for women over time, and unsuccessful attempts turning into a **chronic unhappiness**. Consequently, the reactions developed at and after the diagnosis drag women into both a psychological collapse and a vulnerable position against social expectations. Infertility can lead to a decrease in the sense of individuality by driving the individual to **conform** to the expectation of becoming a mother without critical thought. In this context, as Remennick (2000), states, infertility becomes a **“dominant status”** (*master status*) that overshadows all other qualities of the woman, and the self begins to be perceived merely as a **“body undergoing treatment.”**

The study findings indicate that social support in the infertility process is a **complex realm of experience** for women, where support from the spouse and family is perceived as a resource, while the broader social circle is frequently perceived as a **stressor**. Participants' statements about being overwhelmed by the curious questions and pressure from their social environment align with the risk of **social isolation** and **stigmatisation** experienced by infertile women in the literature. Remennick (2000), states that in pronatalist societies, the style of communication is often so **“non-boundary-setting”** that it **violates privacy**, with women constantly subjected to the question, “When?” This situation explains why participants distance themselves from their social circle and **focused solely on the support of the spouse and nuclear family**. Indeed, (Yılmaz & Yeşiltepe Oskay, 2015), emphasise that family and spousal support are the most **crucial resources** for coping during this process. However, the treatment process is also a **challenging test** that strains marital relationships. Infertility can challenge couples' **coping skills** by leading to **deterioration in their sexual life** and marital relationships (Keskin & Gümüş, 2014). Conversely, high perceived social support positively affects marital adjustment (Bodur and Çoşar, 2013), and providing information about couple communication facilitates process management (Deliktaş & Kızılkaya Beji, 2012). As Ülkar (2022), states, social support can only become a **healing remedy** when it directly addresses the individual's needs and the source of stress (e.g., non-judgmental listening or accurate information). In this context, the participants' most concrete and **unmet expectations** are seen to be directed toward healthcare personnel. The finding that women in the study primarily expect **“cheerfulness, courtesy, patience, and explanatory information”** from healthcare personnel can be read as a reaction to the feeling of **“objectification”** experienced during the treatment process. The vast majority of patients seeking treatment are individuals who have been emotionally and financially exhausted, are experiencing hopelessness, and whose trust has been shaken for a long time. Remennick (2000), states that the treatment process turns into a physically and emotionally draining **“full-time job”** for women. In this challenging process, nurses have a **key role**, beyond medical techniques, in emotionally preparing couples, helping them develop realistic expectations, and receiving **“humane”** care (Aşcı & Kızılkaya Beji, 2012). The literature indicates that psychological support for infertile individuals increases treatment performance (Junior et al., 2002) and ensures the continuity of treatment (Sormunen et al., 2020). As emphasised by Tseng et al. (2024), helping patients find meaning and reduce their hopelessness in this challenging process is not only a medical necessity but also an **ethical responsibility**. Therefore, it is vital that healthcare personnel, especially nurses, **restructure** the treatment process not merely as an area where medical procedures are performed, but as a **“care process”** where couples are **psychosocially supported**, listened to non-judgmentally, and informed.

### Conclusion and recommendations

This study demonstrated that the infertility experience is not limited only to a medical process for women; it is a **temporal “biographical disruption”** that begins with pre-diagnostic social acceptances, deepens with the crisis at the moment of diagnosis, and continues with the uncertainties during the treatment process. Influenced by the pronatalist social structure, women **code** motherhood as a **“matter of existence/non-existence”**; this causes them to experience failures during the treatment process as a **loss of self** and **“grief.”** The feeling of **“objectification”** experienced within the healthcare system and **“violations of privacy”** coming from the social circle further complicate this traumatic process.

In line with these findings, the following recommendations can be presented:

1. **For the Finding of Uncertainty and Fear of Recurrence:** During routine follow-up examinations, nurses should ask the patient, “What is the uncertainty concerning your health that occupies your mind the most today?” and provide uninterrupted space for her to articulate her concerns.
2. **For the Finding of the Altered and Damaged Body:** During physical care or wound dressing, midwives or nurses should gently encourage the patient to look at the wound in order to facilitate reconciliation with her body; in these moments, they should offer verbal affirmation and support for her bodily change, for instance by holding her hand or maintaining eye contact.
3. **For the Finding of the Redefinition of Relationships:** During clinical consultations, physicians and nurses should invite the patient’s spouse or caregiver into the room and ask, “How has the illness process affected your communication?” In doing so, they should observe how couples support one another within the clinical setting and, when necessary, refer them to professional family counselling.
4. **For the Finding of Loneliness and the Need for Support:** At each follow-up appointment, healthcare professionals should use the question, “How lonely have you felt over the past week?” as a screening tool to assess social isolation and provide high-risk patients with a concrete contact list directing them to peer support groups composed of women with similar experiences.

## Scientific contributions and limitations of the study

The results of this phenomenological study make significant contributions to the body of knowledge regarding the infertility experience in Türkiye.

### Scientific contributions

- **Depth and Integrity:** The study offers an in-depth perspective that handles infertility not just as a medical condition, but within a temporal whole encompassing **pre-diagnostic construals, the crisis at the moment of diagnosis, and socio-cultural pressures during the treatment process.** This strengthens the literature that approaches the topic as a biographical disruption.
- **Cultural Context:** It adds the subjective experiences of women in a **pronatalist and traditional social structure**, where motherhood is coded with metaphors of “gaining existence” and “dominance,” providing a unique cultural context to the existing international literature.
- **Practice-Oriented Implications:** Findings regarding the expectation of **“humane care”** and the feeling of **“objectification”** from healthcare personnel offer concrete guidance for nursing and psychological counselling practices.
- **Social Support Paradox:** By detailing the paradox where the social circle is a source of pressure while the spouse and nuclear family are sources of support, the study emphasises the need for infertility counselling to focus on social network management.

### Limitations

- **Sample Type:** Due to the nature of the phenomenological method, the study focuses only on the **subjective experiences** of women undergoing infertility treatment. The findings cannot be generalised to a broader population.
- **Spousal View:** Only female participants were included in the study. To address infertility as a **couple's experience**, additional studies including the experiences of spouses (men) are necessary.
- **Duration of Diagnosis:** The infertility processes and treatment stages of the participants vary. This heterogeneity may make cross-comparison between experiences challenging.

## Supplementary Material

Supplementary Material COREQ Checklist.docx

## Data Availability

The data supporting the findings of this study have not been made publicly accessible due to the sensitive nature of the research topic and the necessity of protecting participant privacy/confidentiality. Data may be obtained from the responsible author upon reasonable request and under the condition that the necessary ethical permissions are secured.
