# Supplementary material for: “Temporal meaning-making of women's emotional experiences during the infertility treatment process: a qualitative study”
Source: Int J Qual Stud Health Well-being. 2026 May 12;21(1):2672214. doi: 10.1080/17482631.2026.2672214 (PMC13173580; doi:10.1080/17482631.2026.2672214)
Supplement: Supplementary Material — COREQ Checklist.docx [file ZQHW_A_2672214_SM4513.docx]

**COREQ Checklist**

| **Domain 1: Research Team and Reflexivity** | | | | |
| --- | --- | --- | --- | --- |
| **No** | **COREQ Item** | **Corresponding Description in Manuscript** | **Section** | **Page No** |
| **1.** | Interviewer/facilitator | Interviews were conducted by the researchers. | Data Collection | 5 |
| **2.** | Credentials | The researchers hold the titles of Professor and Assistant Professor; however, these were not specified in the manuscript to maintain anonymity. | **---** | **---** |
| **3.** | Occupation | Academicians specializing in the field of public/women's health.  Not included in the manuscript as it is currently anonymized. | **---** | **---** |
| **4.** | Gender | Female/Not explicitly stated in the manuscript . | --- | Not Stated |
| **5.** | Experience and training | Expertise in phenomenological designs and clinical practice. | Data Collection | 5 |
| **6.** | Relationship established | Information provided before interviews to establish rapport. | Ethical Considerations | 6-13 |
| **7.** | Participant knowledge | Participants were informed about the study's purpose and goals. | Ethical Considerations | 6-13 |
| **8.** | Interviewer characteristics | *Epoché* (bracketing), bridling, and reflexive journals were used. | Data Collection / Analysis | 5 |
| **Domain 2: Study Design** | | | | |
| **9.** | Theoretical framework | Descriptive phenomenology (Husserl/Giorgi) was utilized. | Method | 3 |
| **10.** | Participant selection | Purposive and snowball sampling methods were employed. | Method (Participants and Sampling) | 1- 4 |
| **11.** | Method of approach | Initial recruitment via healthcare institution followed by social network outreach. | Method (Participants and Sampling) | 1, 4 |
| **12.** | Sample size | A total of 19 women were included in the study. | Method (Participants and Sampling) | 4 |
| **13.** | Non-participation | Women who were undergoing infertility treatment and agreed to participate in the study were included. | Method (Participants and Sampling) | 4 |
| **14.** | Setting of data collection | Conducted in comfortable settings such as homes, cafes, or isolated hospital rooms. | Method (Data Collection) | 5 |
| **15.** | Presence of non-participants | Not specified whether others were present during the interviews. | --- | Not Stated |
| **16.** | Description of sample | Demographics including age, marriage duration, education, and income were provided. | Results | 8 |
| **17.** | Interview guide | Semi-structured guide covering four temporal dimensions was used. | Method (Data Collection) | 1, 5 |
| **18.** | Repeat interviews | There is no mention of repeat interviews being conducted. | --- | Not Stated |
| **19.** | Audio/visual recording | Interviews were audio-recorded with participant consent. | Method (Data Collection) | 5 |
| **20.** | Field notes | Reflexive journals and the "bridling" method were utilized. | Method (Data Collection/Analysis) | 5- 6 |
| **21.** | Duration | Although the duration of each interview varied depending on the participant, the average length was approximately one hour. | Method (Data Collection) | 5 |
| **22.** | Data saturation | Recruitment stopped upon reaching data saturation and "information power". | Method (Participants and Sampling) | 4 |
| **23.** | Transcripts returned | To maintain strict participant anonymity and confidentiality, contact information was not recorded; thus, transcripts were not returned for comment. | Method (Ethical Considerations) | 7 |
| **Domain 3: Analysis and Findings** | | | | |
| **24.** | Data coding | Data analysis was conducted by the researcher using Giorgi's descriptive phenomenological method. | Method (Data Analysis) | 5-6 |
| **25.** | Description of coding tree | Structural constituents, psychological transformations, and meaning units are presented as a sample cross-section in Table 1. | Results | 7-8 |
| **26.** | Derivation of themes | While a temporal framework (pre-diagnosis, diagnosis, treatment, and future) was established via semi-structured questions, the structural constituents and psychological essences within this framework emerged inductively from participant narratives rather than from researcher pre-construction | Method (Data Analysis) / Results | 6-8 |
| **27.** | Software | No specific software was mentioned for data management or analysis . | **---** | Not Stated |
| **28.** | Participant checking | Due to the participant confidentiality and anonymity protocol (as no contact information was retained), the findings and themes identified at the conclusion of the study were not returned to the participants for member checking. | **---** | Not Stated |
| **29.** | Quotations presented | Findings are supported by rich, direct quotations with each participant identified by a unique code (e.g., P1, P3). | Results | 10-20 |
| **30.** | Consistency between data and findings | There is clear consistency between presented participant data (quotations) and the structural findings. | Results / Trustworthiness | 7, 8 |
| **31.** | Clarity of major themes | Major structural constituents within the temporal context (Past, Present, Future) are presented with clarity. | Results | 8 |
| **32.** | Clarity of minor themes | Diverse emotional experiences and temporal meaning shifts among participants are detailed throughout the findings. | Results | 8 |
